# Role of depression-mediated alterations in pro-inflammatory cytokines in carcinogenesis

**DOI:** 10.3389/fimmu.2025.1659153

**Published:** 2025-09-10

**Authors:** Rui Cao, Heguo Jiang, Ping Chen, Chunhua Dai

**Affiliations:** ^1^ Department of Respiratory and Critical Care Medicine Discipline, The Affiliated Hospital of Jiangsu University, Zhenjiang, Jiangsu, China; ^2^ Department of Thoracic Oncology, The Affiliated Hospital of Jiangsu University, Zhenjiang, Jiangsu, China

**Keywords:** cancer, depression, IL-6, TNF-α, IL-1β

## Abstract

This review systematically examines the bidirectional relationship between MDD and cancer, establishing chronic inflammation—orchestrated primarily by IL - 6, TNF-α, and IL - 1β—as a critical biological link. It delineates the pathophysiological mechanisms through which depression activates the HPA axis, induces gut microbiota disruption, and provokes immune dysregulation, collectively fostering a systemic pro-inflammatory state. This state further potentiates oncogenesis via sustained activation of key signaling pathways—including JAK-STAT3, NF-κB, MAPK, and PI3K/AKT—that drive tumor proliferation, angiogenesis, immune evasion, and metastatic behavior. Although preclinical evidence is robust, clinical translation remains impeded by significant heterogeneity in inflammatory biomarkers—particularly IL - 1β—and a stark deficiency of rigorous interventional trials evaluating anti-cytokine biologics in cancer patients with comorbid depression. Future research must therefore advance beyond mechanistic inquiry toward inflammation-based patient stratification and prospective interventional studies, aiming to validate multimodal strategies targeting the depression-inflammation-cancer axis and ultimately propel psycho-oncology into an era of precision medicine.

## 1 Introduction

Cancer represents a preeminent cause of global mortality (1). Carcinogenesis demonstrates a robust association with an array of established risk determinants—including tobacco consumption, dietary patterns, occupational exposures, and genetic predisposition—each significantly elevating disease susceptibility (2).

Throughout the disease trajectory—spanning diagnosis, therapeutic intervention, convalescence, potential recurrence, and terminal phases—malignancy patients confront significant psychological sequelae, establishing this population as a critical focus for targeted psychological support (3). A cancer diagnosis substantially predisposes individuals to depressive pathology; empirical investigations reveal a fourfold greater prevalence of depression among oncology patients relative to the general populace (4), with aggregate global incidence approximating 33.16% (5). Critically, the manifestation of depressive symptomatology correlates with heightened hazards of disease recurrence and mortality (6).

Major Depressive Disorder (MDD) manifests characteristic features of chronic, low-grade systemic inflammation, incorporating both neuroinflammatory processes and dysregulated circulatory inflammatory mediators (7). Circulatory inflammation, in particular, assumes considerable pathophysiological significance in oncological progression. Depressive states induce persistent activation of the Hypothalamic-Pituitary-Adrenal (HPA) axis, directly facilitating neoplastic advancement. Concomitant HPA axis dysregulation further amplifies pro-inflammatory cytokine production, thereby exerting analogous oncogenic effects (8). Additionally, depressive pathology directly engages the inflammatory response system (IRS), precipitating immune dysregulation. This is evidenced by upregulated inflammatory gene expression within hematological lineages and, notably, elevated serum concentrations of pro-inflammatory cytokines (9).

Thus, depressive pathophysiology perturbs cytokine homeostasis through direct and indirect augmentation of pro-inflammatory mediators. This dysregulation subsequently facilitates cardinal procancerous pathways: sustaining aberrant proliferative signaling, circumventing apoptotic mechanisms, evading immune surveillance, facilitating angiogenesis, and driving invasive and metastatic processes (10). Collectively, these mechanisms accelerate disease progression. Consequently, a complex pathophysiological interplay exists between depression and carcinogenesis, wherein dysregulation of circulating pro-inflammatory cytokines constitutes a pivotal mechanistic nexus.

This review systematically synthesizes current evidence elucidating cytokine disturbances consequent to depressive disorders and their mechanistic contributions to oncogenesis and progression. It examines how depression, via dysregulation of pro-inflammatory cytokine networks, potentiates cancer pathogenesis and advancement, thereby underscoring the clinical imperative for enhanced focus on the psychological well-being of cancer patients.

### 2.1 MDD and neuroinflammation

Neuroinflammation constitutes an immune response to central nervous system (CNS) injury, characterized principally by microglial and astrocytic activation alongside infiltration of peripheral immune cells (11). Within depression models, sustained microglial activation accompanied by elevated pro-inflammatory mediators is characteristically observed. Microglial priming is critically orchestrated through Toll-like receptors (TLRs; notably TLR4): TLR4 binding to its agonist initiates myeloid differentiation primary response 88 (MyD88)-dependent signaling, inducing interleukin-1 receptor-associated kinase (IRAK) autophosphorylation. Following dissociation of phosphorylated IRAK from MyD88, phospho-IRAK interacts with TNF Receptor-Associated Factor 6 (TRAF6), thereby activating the Transforming Growth Factor Beta-Activated Kinase 1 (TAK1) complex. This complex subsequently propagates two principal inflammatory signaling cascades: the Mitogen-Activated Protein Kinase (MAPK) pathway and the nuclear factor-κB (NF-κB) pathway. These cascades collectively drive:expression of pro-inflammatory genes (including tumor necrosis factor-alpha [TNF-α], interleukin-6 [IL - 6], and pro-interleukin-1β [pro-IL-1β, the precursor to interleukin-1β]) and activation of the nucleotide-binding oligomerization domain-like receptor family pyrin domain-containing 3 (NLRP3) inflammasome (12).

Concomitantly, peripheral inflammatory mediators—such as IL - 1β, IL - 6, TNF-α, interferon-gamma (IFN-γ), and chemokine (C-C motif) ligand 2 (CCL2)—compromise blood-brain barrier (BBB) integrity, increasing its permeability. This facilitates parenchymal infiltration of both these mediators and peripheral immune cells. Such infiltration not only directly potentiates CNS inflammation but also further stimulates microglial activation. Activated microglia, in turn, secrete copious cytokines and chemokines, propagating a self-reinforcing inflammatory cycle that perpetuates and intensifies the neuropathological process (13).

### 2.2 MDD and peripheral inflammation

Peripheral inflammation is the body’s protective immune response to extra-central nervous system injury and involves the release of pro-inflammatory cytokines (14). In patients with MDD, chronic stress induces persistent activation of the HPA axis, resulting in sustained hypercortisolemia. Elevated cortisol levels impair glucocorticoid receptor (GR) functionality within immune cells, attenuating their inhibitory regulation of NF-κB and thereby facilitating continuous release of pro-inflammatory mediators (15).

Concurrently, significant immune cell dysregulation is observed: circulating pro-inflammatory Ly6C^+^ monocytes and neutrophils are elevated, secreting IL - 6, TNF-α, and matrix metalloproteinase 8 (MMP8), which collectively compromise vascular endothelial integrity and increase BBB permeability (16). Furthermore, an expansion of T helper 17 (Th17) cells—characterized by interleukin-17 (IL - 17) secretion—alongside a reduction in regulatory T cells (Tregs), culminates in loss of immunosuppressive capacity, thereby instigating a persistent systemic inflammatory state (17).

Additionally, mitochondrial dysfunction induces excessive generation of reactive oxygen species (ROS), causing oxidative damage to mitochondrial DNA (mtDNA). The subsequent release of damaged mtDNA into systemic circulation upregulates IL - 6 and TNF-α expression via activation of the Toll-like receptor 9 (TLR9)/NF-κB pathway (17). Gut microbiota dysbiosis, exemplified by increased Enterococcus spp. abundance, disrupts intestinal barrier function and facilitates translocation of lipopolysaccharide (LPS) into the bloodstream, thereby activating the TLR4/NF-κB signaling cascade (13).

Collectively, these interconnected mechanisms drive the sustained systemic inflammatory state characteristic of MDD patients.

## 3 Relationship between MDD and cancer, carcinogenic process of MDD

A robust bidirectional association exists between MDD and cancer. Epidemiological studies indicate that MDD elevates overall cancer incidence by 22% (18) and significantly increases mortality risk across multiple cancer types—colorectal cancer (83%), lung cancer (59%), prostate cancer (74%), and breast cancer (23%)—with meta-analyses demonstrating a 38% increase in overall cancer-related mortality among MDD patients (19). Conversely, cancer diagnosis substantially predisposes individuals to depression; for instance, the prevalence of depression among lung cancer patients reaches as high as 57.1% (20).

The underlying biological mechanisms primarily involve dysregulation of neuroendocrine and immuno-inflammatory pathways:1)Metabolic Reprogramming: MDD suppresses mitochondrial oxidative phosphorylation (OXPHOS) and activates glycolytic metabolism in tumors, concurrent with upregulation of the glutamine transporter SLC38A2. These alterations collectively facilitate tumor proliferation and progression (21).2)Sympathetic Activation: Activation of the sympathetic nervous system releases norepinephrine (NE), which binds to β2-adrenergic receptors (β2AR) on tumor cells and induces secretion of neuropeptide Y (NPY). This promotes recruitment of myeloid-derived suppressor cells (MDSCs) and their differentiation into tumor-associated macrophages (TAMs), ultimately fueling tumor progression via the IL - 6/transcription 3(IL - 6/STAT3) signaling axis (22).3)Inflammation and Epigenetic Regulation: Hyperactivity of the HPA axis and GR resistance attenuate suppression of NF-κB, resulting in sustained release of pro-inflammatory cytokines (e.g., IL - 6, TNF-α) that enhance tumor cell survival. Concurrently, NE/β2AR signaling inhibits the demethylase ALKBH5, increasing RNA m^6^A methylation and thereby promoting self-renewal of cancer stem cells (23).Together, these mechanisms constitute a vicious cycle through which MDD accelerates tumorigenesis and disease progression.

## 4 Important abnormal cytokines associated with MDD

Cytokines are key soluble mediators of immune and inflammatory responses, regulating immune cell function via specific receptor binding (24). MDD is characterized by broad cytokine dysregulation, including elevated levels of IL - 1β, IL - 2, IL - 4, IL - 6, IL - 8, IL - 10, IL - 12, IL - 13, IL - 15, and TNF-α, alongside decreased IL - 5, IL - 12 p70, and TNF-β (25). A positive correlation was observed between Hamilton Depression Rating Scale (HAMD) scores and serum IL - 2 levels in female patients with major depressive disorder, indicating that more severe depressive symptoms are associated with higher IL - 2 concentrations—a relationship that demonstrates gender-specificity (26). Plasma IL - 10 is notably elevated and correlates with depression severity (27), while Th17-derived IL - 17 is also increased and weakly associated with higher Hamilton Depression Rating Scale (HDRS) scores (28). In contrast, IFN-γ alterations are inconsistent and insignificant in untreated adolescents, likely reflecting disease heterogeneity (29). A summary of cytokine changes in cancer with depression: human and animal studies is provided in **Table 1**.

**Table 1:** Cytokine changes in cancer with depression: a summary of human and animal studies.

Study type	Cancer type	Depression assessment method	Major cytokine/molecular findings
Human	Breast Cancer	Patient Health Questionnaire 9 (PHQ - 9)	Levels of IL - 6, IL - 10, IL - 17, IL - 21, IL - 23, IL - 35, and CRP were demonstrated to be significantly elevated (106).
	Lung Cancer	Hospital Anxiety and Depression Scale (HADS)	TNF-α, IL - 1β, IL - 6, and IL - 17 each demonstrated significant positive correlations with HADS-A scores (P < 0.05), the prevalence of anxiety (P < 0.05), HADS-D scores (P < 0.05), and the incidence of depression (P < 0.05 for all) (107).
	Colorectal Cancer	HADS	Anxiety and/or depression demonstrated a significant positive correlation with IL - 1β, IL - 6, IL - 8, and TNF-α, whereas an inverse correlation was observed with IL - 10 (108).
	Pancreatic Cancer	HDRS	Individuals with depression exhibited significantly elevated levels of IL - 6 and a reduced IL - 2/IL-4 ratio compared to non-depressed participants (109).
Animal	Breast cancer (4T1 model)	Chronic Restraint Stress (CRS)	Levels of IFN-γ, IL - 1β, IL - 4, IL - 6, IL - 8, and TNF-α were observed to be elevated, whereas concentrations of IL - 2 and IL - 10 were concurrently reduced (110).
	Prostate Cancer	Chronic Unpredictable Mild Stress (CUMS)	Among prostate cancer patients with elevated psychological depression scores, a significant increase was observed in macrophage infiltration as well as elevated expression of NPY and IL - 6 (22).

**Figure 1:**
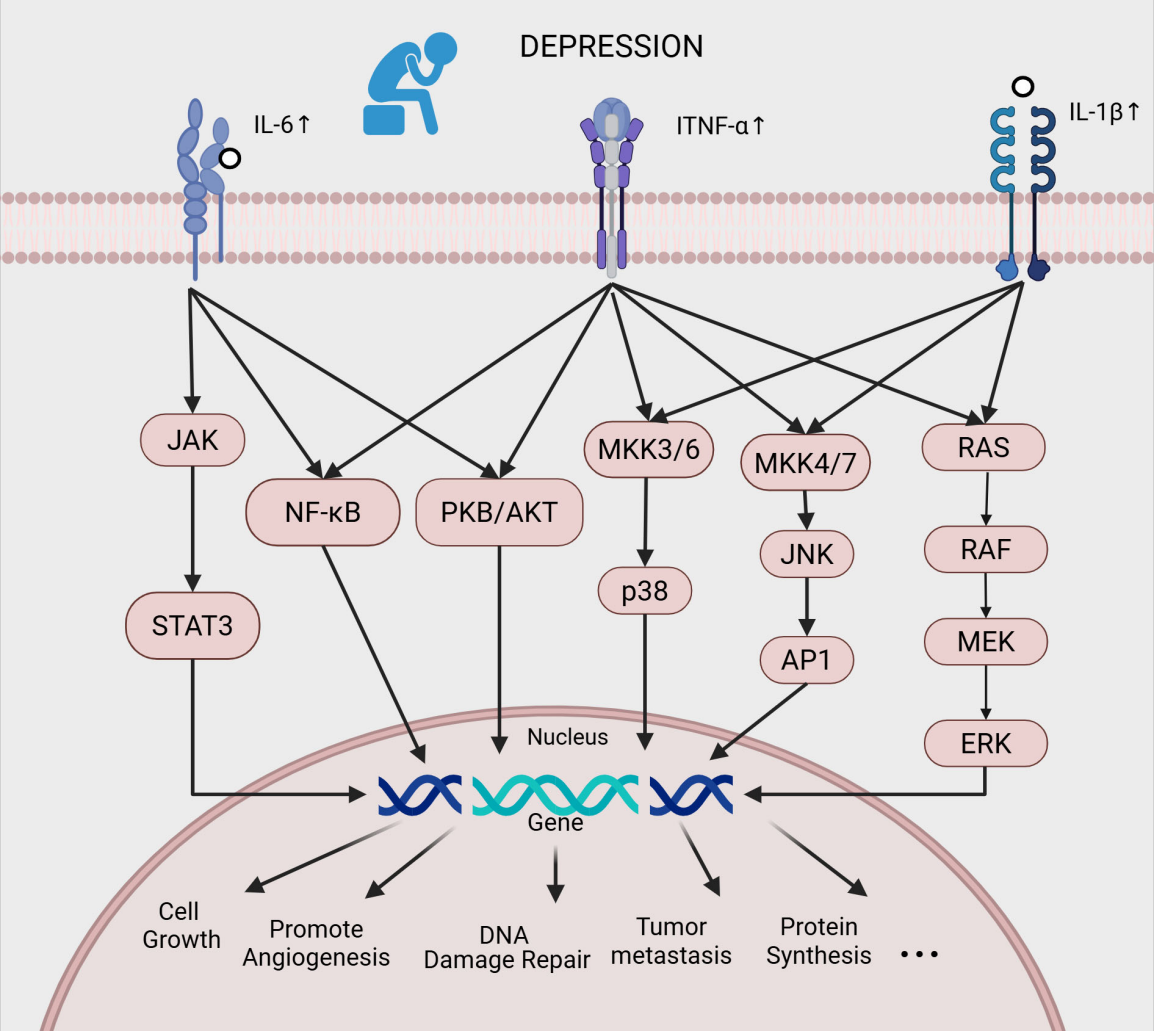
Depression constitutes a molecular bridge to depressive co-morbid tumors by up-regulating inflammatory factors such as IL - 6, TNF-α, and IL - 1β, activating multiple intracellular signaling pathways (JAK, NF-κB, MAPK, and PI3K/AKT), which in turn drive changes in gene expression and promote tumor growth, angiogenesis, metastasis, and metabolic reorganization.).

This review summarizes changes in three major pro-inflammatory cytokines—IL-6, TNF-α, and IL - 1β—in MDD and their influence on cancer progression. These cytokines are particularly representative due to their consistently elevated pro-inflammatory activity in MDD and strong links to neuroinflammation and systemic pathology. Their dysregulation not only contributes to depression but may also promote cancer via downstream inflammatory signaling, thereby serving as critical molecular intermediaries between depression and tumor progression. The mechanism of depression-promoted tumor progression via IL-6/TNF-α/IL-1β upregulation and activation of JAK/STAT, NF-κB, MAPK, and PI3K/AKT pathways is summarized in **Figure 1**.

### 4.1 Depression leads to changes in IL - 6 levels

IL-6 is a pleiotropic pro-inflammatory cytokine whose biological functions depend on the formation of a complex comprising IL - 6, either membrane-bound or soluble IL - 6 receptor (mIL-6R/sIL-6R), and the signal-transducing subunit glycoprotein 130 (gp130), through which it activates downstream signaling pathways to exert its biochemical effects (30). In the context of depression, significant alterations in both central and peripheral IL - 6 levels have been observed. Consistent clinical evidence indicates elevated IL - 6 concentrations in the peripheral blood, serum, plasma, and cerebrospinal fluid of individuals with MDD, with these levels positively correlating with depression severity and disease progression{sp} (25, 31 – 33){/sp}.Furthermore, treatment with selective serotonin reuptake inhibitors (SSRIs) has been associated with a reduction in circulating IL - 6 levels in some patients (34), suggesting that antidepressant therapy may partially reverse this inflammatory state.

These findings establish a clear link between IL - 6 and depressive pathology; however, existing evidence remains limited. Most conclusions are derived from cross-sectional studies, and there is a scarcity of large-scale longitudinal cohort data to substantiate a causal relationship between IL - 6 dynamics and the course of depression. Although SSRIs may reduce IL - 6 in certain cases, the antidepressant efficacy of directly targeting the IL - 6 receptor—such as with tocilizumab—has not been conclusively demonstrated in human studies, despite promising results in animal models and limited clinical translation (35).

Depression is associated with expanded monocyte and neutrophil populations. MDD directly activates monocytes and macrophages, enhancing IL - 6 production (36). Depressive states also trigger HPA axis activation, increasing glucocorticoid secretion. While glucocorticoids typically exert protective effects through GR binding, chronic HPA overactivity causes sustained glucocorticoid elevation, inducing GR resistance and impairing GR function in immune cells. This disrupts IL - 6 suppression, further elevating IL - 6 levels (7).

The gut microbiota in depressed patients also shows distinct dysbiosis relative to healthy individuals, marked by reduced diversity, compositional changes, expansion of pro-inflammatory bacteria, and loss of anti-inflammatory taxa. These shifts induce expression of mucosal inflammatory markers like TLR4, damage the gut barrier, and raise permeability, enabling endotoxins such as LPS to enter the bloodstream. This triggers immune activation and inflammatory cytokine release, including IL - 6 (37, 38).

In summary, elevated IL - 6 and its mechanistic links to depression reinforce the inflammatory hypothesis of the disorder and suggest anti-inflammatory therapy as a promising direction. However, clinical translation remains limited: current evidence relies largely on cross-sectional data, impeding causal conclusions about IL - 6 in depression. Although SSRIs may lower IL - 6 in some patients, direct IL - 6 pathway inhibition—such as with tocilizumab—has not demonstrated robust antidepressant efficacy in human studies, highlighting persistent translational barriers. Large longitudinal studies are urgently needed to establish causality and advance personalized anti-inflammatory treatment strategies, including predictive biomarkers of response.

### 4.2 Possible mechanisms by which depression promotes cancer progression through elevated IL - 6

The IL - 6–IL-6R–gp130 complex activates Janus Kinases (JAKs), thereby initiating multiple downstream signaling cascades. The first is the JAK–STAT3 pathway, wherein JAK induces autophosphorylation, facilitating dimerization and nuclear translocation of the signal transducer and activator of STAT3, ultimately mediating intracellular signal transduction and regulating gene expression (39).The second involves the MAPK/Extracellular Signal-Regulated Kinase (ERK) pathway: JAK activates the RAS–RAF cascade, resulting in hyperphosphorylation of MAPK and promoting cellular proliferation (40).The third entails the Phosphoinositide 3-Kinase–Protein Kinase B (PI3K–AKT) pathway, through which JAK phosphorylates and activates PI3K, converting phosphatidylinositol 4,5-bisphosphate (PIP2) to phosphatidylinositol (3–5)-trisphosphate (PIP3). PIP3 in turn phosphorylates and activates AKT, a regulator of cell survival (41).

**Figure 2:**
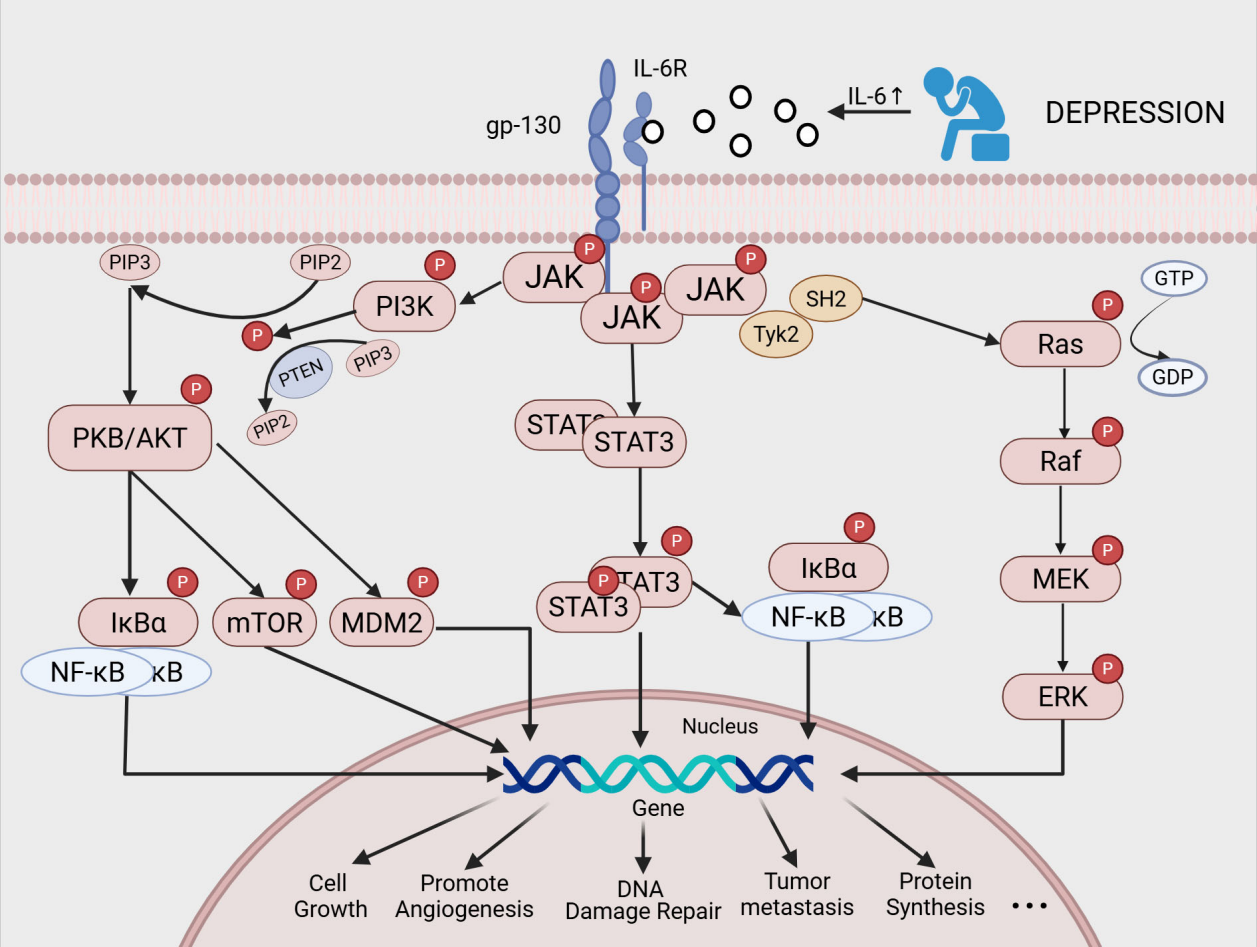
Possible mechanisms by which a depressive mood can promote cancer progression through increased levels of IL - 6:The IL - 6-IL-6R-gp130 complex activates JAK kinases, which regulate the expression of multiple genes through the JAK-STAT3, MAPK/ERK, and PI3K-PKB/AKT pathways. These pathways mediate tumor biological processes, such as cell proliferation, differentiation, and metastasis. They also promote the formation of the tumor microenvironment and enhance the survival and proliferation capabilities of tumor cells.).

Chronic inflammation associated with depression may elevate IL - 6 levels, thereby modulating the expression of various genes involved in cellular proliferation, differentiation, and metastasis. These processes contribute to the formation of a tumor-promoting microenvironment and enhance the survival and proliferative capacity of cancer cells. The precise mechanisms by which IL-6 facilitates tumor biological processes through downstream signaling are illustrated in **Figure 2**.

#### 4.2.1 Relationship between IL - 6 and the JAK-STAT3 signaling pathway

Under physiological conditions, STAT3 signaling is tightly regulated in healthy cells, however, in malignant cells—particularly in lung cancer—STAT3 is frequently and constitutively activated, contributing to the modulation of the tumor microenvironment. IL - 6 serves as a critical activator of STAT3 and establishes a forward-feedback loop via the IL - 6–JAK–STAT3 axis: paracrine IL - 6 binding to IL - 6R–gp130 complexes induces STAT3 activation, which in turn stimulates autocrine IL - 6 expression within cancer cells (42). Activated STAT3 promotes cellular proliferation by upregulating anti-apoptotic genes such as B-Cell Lymphoma 2(BCL - 2) and cell cycle regulators including MYelocytomatosis oncogene(MYC) (43). It further enhances the expression of cytokines and pro-angiogenic factors such as Vascular endothelial growth factor(VEGF) and IL - 1β, thereby fostering an immunosuppressive microenvironment and facilitating angiogenesis (44). STAT3 also sustains constitutive NF-κB activity in tumor cells, prolonging its nuclear retention and amplifying cancer-associated NF-κB signaling, which collectively induces the expression of genes implicated in proliferation, anti-apoptosis, angiogenesis, and metastasis (45). In depressed patients, elevated IL - 6 levels lead to hyperactivation of STAT3. Through the JAK-STAT3 signaling cascade, this exacerbates tumor development by dysregulating the cell cycle, promoting angiogenesis, and supporting other pro-oncogenic processes.

#### 4.2.2 Relationship between IL - 6 and the MAPK/ERK signaling pathway

The IL - 6–IL-6R–gp130 complex initiates JAK activation, which in turn phosphorylates and activates SRC homology 2 domain-containing protein tyrosine phosphatase 2 (SHP2), thereby facilitating the stimulation of the Rat sarcoma virus (RAS)/Rapidly Accelerated Fibrosarcoma (RAF)/Mitogen-activated protein kinase kinase (MEK)/ERK signaling cascade (46). Elevated IL - 6 levels observed in cancer patients with depressive symptoms may promote oncogenesis through this pathway. Sustained activation of the MEK/ERK signaling axis is capable of transforming normal cells into a malignant phenotype, whereas inhibition of this pathway can revert tumor cells to a non-transformed state *in vitro* and effectively suppress tumor growth *in vivo (* 47).The MAPK/ERK pathway also contributes to the regulation of the cell cycle, particularly during the transition from G1 to S phase (48). Additionally, its activation upregulates VEGF expression in tumor-associated endothelial cells, thereby influencing angiogenic processes (49).Thus, depression-associated elevation of IL - 6 activates the MAPK/ERK pathway, influencing tumor progression through diverse mechanisms including cell proliferation, cell cycle regulation, and angiogenesis.

#### 4.2.3 Relationship between IL - 6 and the PI3K- Akt signaling pathway

IL-6 additionally mediates its effects through the PI3K–Akt signaling cascade. Upon activation, AKT phosphorylates the cytoplasmic protein Mouse Double Minute 2 Homolog(MDM2), facilitating its nuclear translocation. Within the nucleus, MDM2 binds Tumor Protein P53 (TP53) and promotes its ubiquitination, leading to proteasomal degradation. This process impairs p53-dependent functions—including growth inhibition, induction of cellular senescence, and apoptosis in response to DNA damage and oncogenic stress—thereby facilitating uncontrolled cellular proliferation (50).Furthermore, this pathway contributes to oncogenesis through multiple mechanisms: it activates oncoproteins such as MYC and PDK1; phosphorylates and inactivates the checkpoint kinase CHK1; and suppresses the transcription of tumor suppressors including B-Cell Lymphoma-extra Large(BCL-xL)/BCL-2 and p130/RB2. These actions collectively dysregulate cell cycle control and enhance cellular proliferation and survival (51). Additionally, PI3K/AKT signaling stimulates protein synthesis and cell growth via activation of the mammalian target of rapamycin (mTOR) (52), and promotes cell survival through NF-κB activation (53).Consequently, elevated IL - 6 levels associated with depressive states induce hyperactivation of the PI3K–AKT pathway, thereby substantively contributing to tumor progression.

Depressive states markedly elevate both peripheral and central IL - 6 levels through multiple mechanisms: immune cell activation, hyperactivation of the HPA axis culminating in glucocorticoid receptor resistance, and endotoxemia resulting from gut barrier disruption. Elevated IL - 6, in turn, acts via the IL - 6R/gp130 complex to synergistically promote tumor cell proliferation, angiogenesis, immune evasion, and metastatic processes through the constitutive activation of JAK–STAT3, MAPK/ERK, and PI3K–AKT signaling cascades.

Although certain antidepressant interventions can partially attenuate IL - 6 upregulation, the role of IL - 6 as a central mediator in depression-associated cancer progression remains to be conclusively established through large-scale longitudinal studies. Furthermore, the therapeutic potential of strategies specifically targeting IL - 6 signaling warrants more extensive investigation to assess its clinical applicability.

### 4.3 Depression leads to changes in TNF-α levels

TNF-α is a pleiotropic cytokine that orchestrates a dual role in cancer progression by modulating both apoptotic and proliferative pathways (54). Consistent clinical evidence indicates that patients with MDD exhibit significantly elevated serum concentrations of TNF-α compared to healthy controls, with levels positively correlating with HAMD scores (55, 56). Treatment with SSRIs has been shown to substantially reduce TNF-α levels, suggesting a partial amelioration of the inflammatory state (34).

In preclinical studies, anti-TNF-α monoclonal antibodies (e.g., infliximab) demonstrate rapid antidepressant effects in murine models, including behavioral improvements, suppression of hippocampal astrocyte activation, and reduced neuronal apoptosis (57). However, corresponding clinical trials in humans have not unequivocally supported their efficacy as monotherapy for depression. This discrepancy may be attributed to limited blood-brain barrier penetration of monoclonal antibodies and the more complex neuroimmune-neurotransmitter interactions inherent to human neurobiology (58).

Hyperactivity of the HPA axis in MDD leads to elevated cortisol levels and subsequent GR resistance, which impairs the negative regulation of TNF-α release and contributes to its increased systemic concentrations (7). Depressive states are frequently accompanied by gut microbiota dysbiosis and upregulation of inflammatory mediators within the intestinal mucosa, resulting in compromised barrier integrity and enhanced permeability. This dysfunction facilitates translocation of LPS into systemic circulation, where it activates peripheral immune cells and stimulates TNF-α secretion (37, 38).Animal studies further demonstrate that LPS administration induces elevated serum TNF-α levels and depression-like behaviors in mice. Pretreatment with SSRIs, such as fluoxetine or paroxetine, effectively attenuates these responses (59).

Elevated TNF-α represents a prominent inflammatory biomarker in major depression, correlating with disease severity and demonstrating partial reversibility following SSRI treatment. Although TNF-α inhibition (e.g., with infliximab) has shown efficacy in animal models, its utility as monotherapy for human depression remains limited. This discrepancy underscores the complexity of translating fundamental inflammatory mechanisms into effective clinical interventions. Key challenges include restricted blood-brain barrier permeability of biologic agents, divergence between human neuroimmune circuitry and animal models, and the multifactorial etiology of depression—encompassing HPA axis dysregulation, gut microbiota disruption, and subsequent LPS translocation.

Future therapeutic strategies must extend beyond singular targets toward integrated, multidimensional approaches that concurrently modulate the HPA–immune–gut–brain axis. Moreover, the potential of anti-inflammatory augmentation therapy warrants rigorous evaluation, particularly in defined inflammatory subtypes of depressed patients.

### 4.4 Possible mechanisms by which depression promotes cancer progression through elevated TNF-α

**Figure 3:**
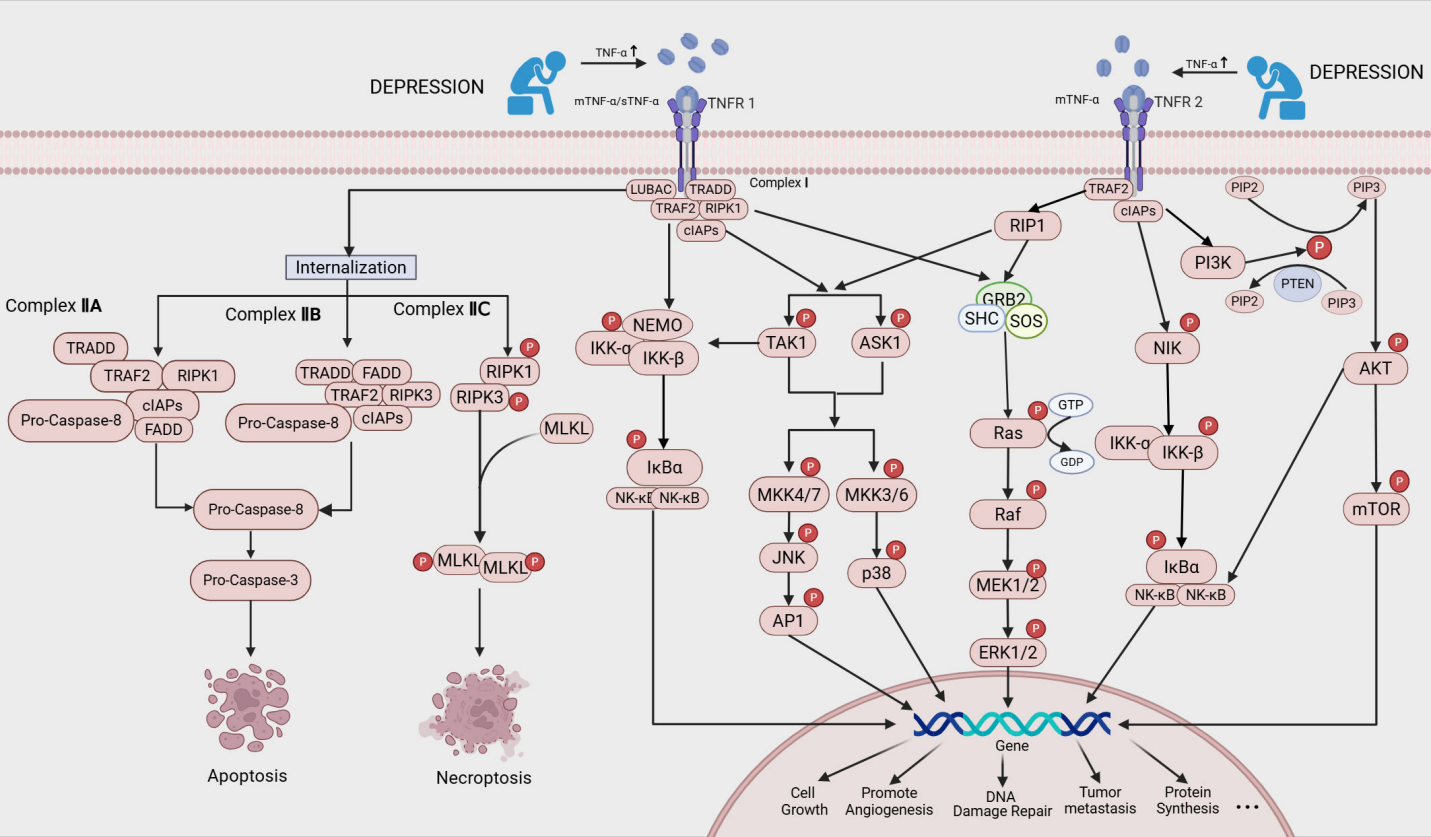
Possible mechanisms by which a depressive mood can promote cancer progression through increased levels of TNF-α:TNF-α plays a dual role in promoting both apoptosis and cell growth. Binding of TNF-α to TNFR1 can lead to the formation of complexes IIA, IIB and IIC from complex I, inducing apoptosis and necrosis. TNF-α also regulates the NF-κB, PI3K/AKT and MAPK pathways, which play a role in inflammation, immune modulation, tumor development and growth.).

TNF-α functions as a transmembrane homotrimeric protein that undergoes proteolytic processing by convertase enzymes, yielding two principal isoforms: membrane-bound (mTNF-α) and soluble (sTNF-α). Its physiological effects are mediated through engagement with distinct receptors, with functional outcomes contingent upon receptor specificity. Binding to tumor necrosis factor receptor 1(TNFR1) enables TNF-α to activate pro-inflammatory gene expression and promote cancer progression via pathways such as NF-κB, while also triggering programmed cell death through both apoptosis and necroptosis (54, 60). In contrast, tumor necrosis factor receptor 2(TNFR2) is primarily associated with cellular proliferation and survival (61). Thus, TNF-α orchestrates pivotal roles in inflammatory responses, immune regulation, and tumorigenesis. The dual role of TNF-α in promoting apoptosis and cell growth, and its regulation of key oncogenic pathways, is illustrated in **Figure 3**.

#### 4.4.1 Relationship between TNF-α and NF-кB signaling pathway

In a state of depression, circulating levels of TNF-α are significantly elevated. Upon binding to TNFR1, TNF-α facilitates the formation of Complex I, which subsequently recruits and activates the IκB kinase (IKK) complex, leading to the activation of the NF-κB signaling pathway. Furthermore, TNF-α promotes the association of cellular Inhibitor of Apoptosis Proteins (cIAPs) with Receptor-Interacting Serine/Threonine-Protein Kinase 1 (RIPK1), thereby recruiting TAK1. TAK1 in turn phosphorylates and activates the IKK complex, resulting in the rapid phosphorylation and ubiquitin-mediated degradation of Inhibitor of kappa B-alpha (IκB-α). This process liberates NF-κB, enabling its translocation into the nucleus, where it initiates the transcription of various response genes, including the anti-apoptotic gene Cellular FLICE-Inhibitory Protein (cFLIP). cFLIP inhibits Caspase-8 activation mediated by Complex IIa, thereby counteracting the apoptotic process (62). It is important to note that TNFR1 induces cell death only under specific conditions (63).

In contrast, TNFR2 possesses no intrinsic catalytic activity and relies on the recruitment of adaptor proteins, primarily TNF Receptor-Associated Factor 2 (TRAF2), for signal transduction. The binding of TNF-α to TNFR2 activates TRAF2, which then associates with NF-κB-Inducing Kinase (NIK). This interaction activates IKK through phosphorylation, triggering downstream signals that promote tumor cell survival and confer resistance to apoptosis (64).

A significant positive feedback loop exists between TNF-α and NF-κB: TNF-α sustains the activation of NF-κB in pre-malignant and cancerous cells in an autocrine and paracrine manner, which in turn induces these cells to highly express TNF-α, forming a self-perpetuating vicious cycle. This loop not only helps establish a pro-survival inflammatory microenvironment but also suppresses anti-cancer immunity, ultimately fostering tumor growth, proliferation, and metastasis (65, 66). Depressive mood, by markedly elevating TNF-α levels, hyperactivates NF-κB signaling, thereby shaping an immunosuppressive tumor microenvironment and inducing immune escape, which consequently accelerates cancer progression.

#### 4.4.2 Relationship between TNF-α and the PI3K/AKT signaling pathway

Under depressive conditions, elevated levels of TNF-α facilitate tumor cell growth and survival through activation of the PI3K–AKT signaling pathway (67). Specifically, upon binding to TNFR2, TNF-α induces phosphorylation of PI3K, which in turn phosphorylates and activates the downstream kinase AKT (68). This signaling cascade not only directly promotes tumor cell proliferation and survival but also upregulates the expression of phosphorylated AKT (p-AKT), thereby triggering nuclear translocation of NF-κB. This process further modulates the transcription of multiple tumor-associated genes and enhances resistance to apoptosis (53). In summary, within the depressive milieu, elevated TNF-α may significantly potentiate tumor cell survival, proliferation, and anti-apoptotic capacity through the PI3K/AKT pathway and its concerted modulation of NF-κB.

#### 4.4.3 Relationship between TNF-α and MAPK signaling pathway

Elevated levels of TNF-α resulting from depressive states effectively activate multiple MAPK signaling pathways—including c-Jun N-terminal Kinase (JNK), ERK, and p38—thereby facilitating tumor progression (69, 70). The underlying mechanisms involve the TNF-α-induced polyubiquitination of Receptor-Interacting Protein 1 (RIP1) through a complex comprising TRAF2 and cellular Inhibitor of Apoptosis Protein 1/2 (cIAP1/2), leading to the assembly of the RIP1 signaling complex (71). This complex subsequently recruits and activates TAK1 and Apoptosis Signal-Regulating Kinase 1 (ASK1), which phosphorylate Mitogen-Activated Protein Kinase Kinase 4/7 (MKK4/7). These in turn activate JNK.

Activated JNK promotes the phosphorylation of the transcription factor Jun, facilitating Activator Protein 1 (AP1) activation. This regulates the expression of genes involved in cell cycle progression, survival, apoptosis, and metalloproteinases, collectively enhancing cell survival (72). In lung cancer models, JNK also suppresses TP53 gene transcription, reduces p53 protein abundance, and consequently inhibits apoptosis while augmenting cisplatin resistance (73).

Concurrently, TAK1 and ASK1 phosphorylate MKK3/6 to activate p38 MAPK (74). p38 induces cell differentiation, mediates anti-apoptotic inflammatory signaling, modulates autophagy, promotes beta-catenin (β-catenin) accumulation, triggers cell cycle arrest, upregulates matrix metalloproteinase expression, and participates in DNA repair processes. It plays a pivotal role in matrix remodeling and degradation during metastatic dissemination (72).

Furthermore, TNF-α activates the RAS/RAF/MEK/ERK signaling cascade through the TRAF2–RIP1/SHC–Growth Factor Receptor-Bound Protein 2 (Grb2)–Son of Sevenless (SOS) axis. This cascade modulates transcription factors such as MYC and E2F, stimulates the expression of cyclins and cyclin-dependent kinases, and promotes VEGF production, collectively orchestrating tumor growth and angiogenesis (75).

Although depression-associated elevation of TNF-α theoretically establishes a molecular foundation for cancer progression—through activation of multiple signaling pathways such as NF-κB, PI3K-AKT, and MAPK that promote inflammatory microenvironments, immune evasion, and apoptosis resistance—its direct clinical translatability faces substantial challenges. Central to these limitations is the fact that these intricately described mechanisms are predominantly derived from *in vitro* and animal models, which inadequately recapitulate the heterogeneity, dynamic adaptation, and compensatory networks of human tumor microenvironments.

Furthermore, TNF-α represents only one component within a broader pro-tumor inflammatory network; monotherapeutic targeting using agents such as infliximab has demonstrated limited efficacy and potential risks in solid tumors. Crucially, there is a lack of robust clinical evidence demonstrating that modulation of depression or direct TNF-α inhibition significantly improves survival outcomes in cancer patients. Future research should prioritize the identification of molecularly defined tumor subtypes reliant on specific inflammatory pathways and cautiously explore integrated management strategies targeting the “depression–immune–tumor axis.”

### 4.5 Depression leads to changes in IL - 1β levels

IL-1β is a central cytokine in innate immunity and inflammatory responses, as well as a key mediator in various pulmonary inflammatory diseases. Its biological activity depends on the formation of a trimeric signaling complex with Type I Interleukin-1 Receptor (IL - 1RI) and the Interleukin-1 Receptor Accessory Protein (IL - 1RAcP), which initiates downstream signaling cascades and drives inflammatory processes (76, 77).

In MDD, findings regarding IL - 1β levels are inconsistent across studies: multiple reports indicate significantly elevated concentrations of IL - 1β in the peripheral blood or serum of patients, positively correlating with depression severity (31, 78). Animal models further demonstrate that LPS-induced activation of the NOD-like receptor family pyrin domain-containing 3 (NLRP3) inflammasome is accompanied by increased IL - 1β (79). However, some meta-analyses show no significant difference in IL - 1β levels between MDD patients and healthy controls (80). These discrepancies may stem from sample heterogeneity (55), methodological variations, or the unique secretory dynamics of IL - 1β—which is released via non-canonical pathways such as exosomal delivery or Gasdermin D pore formation, and is tightly regulated by the extracellular microenvironment. These factors complicate accurate *in vitro* quantification of its *in vivo* bioactivity (81).

Moreover, the pathological effects of IL - 1β are modulated by other biomarkers; for instance, levels of Brain-Derived Neurotrophic Factor (BDNF) significantly influence its impact. When soluble BDNF is low, each 1 pg/mL increase in IL - 1β elevates the risk of antidepressant treatment failure by 17%. In contrast, high BDNF levels completely mitigate this detrimental effect, underscoring the context-dependent contribution of IL - 1β to immunopathology (82).

Depression, as a state of chronic psychological stress, activates the NLRP3 inflammasome through multiple mechanisms. Upon assembly, the inflammasome facilitates the autocleavage of pro-caspase-1 into enzymatically active caspase-1, which in turn cleaves the immature pro-IL-1β into its mature form and promotes its extracellular secretion (83). Furthermore, depression contributes to elevated IL - 1β levels through sustained hyperactivity of the HPA axis (7), aberrant activation of peripheral monocytes and macrophages (36), and disruption of gut microbiota composition (37, 38).

Although several studies support a positive correlation between peripheral IL - 1β levels and both depression severity and lung cancer progression, its clinical utility remains constrained by methodological limitations—such as insufficient sensitivity of current assays to capture non-canonical secretion pathways and bioactive forms of IL - 1β—as well as population heterogeneity, with more pronounced alterations often observed in treatment-resistant depressive subtypes. The biological effects of IL - 1β are highly context-dependent; for instance, higher levels of BDNF can completely counteract its pathological impact, indicating that a single inflammatory marker is insufficient for reliable clinical interpretation.

Future research should focus on developing multidimensional assessment frameworks that integrate inflammatory subtypes, neurotrophic factors, and metabolic parameters. Additionally, targeted investigation into dual-intervention strategies aimed at the IL - 1β signaling pathway in patients with comorbid depression and lung cancer is warranted to transcend the current impasse of “mechanistic clarity amid translational challenges.”

### 4.6 Possible mechanisms by which depression promotes cancer progression through elevated IL - 1β

Depressive states can elevate peripheral blood levels of IL - 1β, a cytokine that plays a critical role in the initiation and progression of malignant tumors. IL - 1β exerts its effects by forming a complex with the IL - 1R1 and its accessory protein IL - 1RAcP. This complex activates both p38 and JNK signaling pathways, leading to the activation of the transcription factor AP1. Concurrently, it stimulates the IKK complex, resulting in the phosphorylation and degradation of Inhibitor of IκB-α, which releases NF-κB and facilitates its nuclear translocation, thereby initiating the transcription of NF-κB-dependent genes (84).

Furthermore, IL - 1β serves as a pivotal driver in the development and metastasis of mesenchymal and epithelial-derived tumors. It induces the acetylation of the mitochondrial inner membrane protein Nicotinamide Nucleotide Transhydrogenase (NNT), enhancing its catalytic activity. This promotes the synthesis of Nicotinamide Adenine Dinucleotide Phosphate (NADPH), maintains iron-sulfur cluster homeostasis, and consequently inhibits ferroptosis in tumor cells (85). IL - 1β also activates the PI3K–AKT signaling pathway, inducing epithelial–mesenchymal transition (EMT) in non-small cell lung cancer (86), and synergistically promotes tumor growth and metastatic processes by stimulating the production of various growth factors, including VEGF (77).

**Figure 4:**
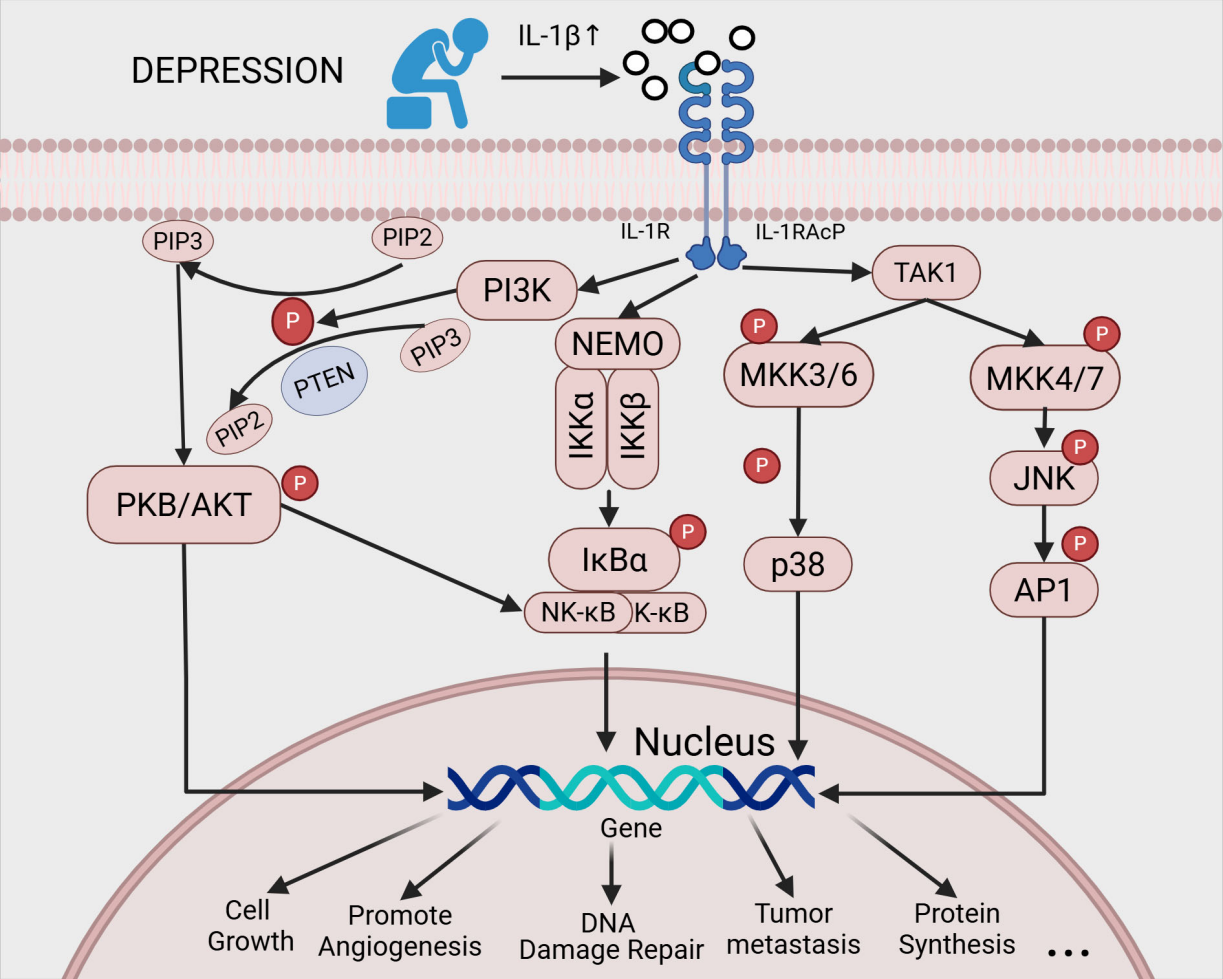
Possible mechanisms by which a depressive mood can promote cancer progression through increased levels of IL - 1β:Following the formation of the IL - 1β/IL-1R/IL-1RAcP complex, p38 and signaling pathways such as JNK, NF-κB and PI3K/AKT are activated, promoting tumor growth and metastasis. This complex plays a key role in the development and progression of malignant tumors.).

Finally, IL - 1β upregulates glycolytic activity in lung adenocarcinoma cells via the p38MAPK pathway, further enhancing their migratory, invasive, and metastatic capabilities (87). The mechanisms by which IL-1β promotes tumor growth and metastasis through downstream signaling cascades are illustrated in **Figure 4**.

Although IL - 1β represents a pivotal theoretical nexus linking depression and cancer, its clinical translation faces dual challenges: while mechanistic studies have confirmed that IL - 1β promotes lung cancer progression through activation of the NF-κB/AP-1 signaling pathways, induction of EMT, and metabolic reprogramming—such as inhibition of ferroptosis and enhancement of glycolysis—its elevation in depressive states exhibits pronounced heterogeneity. This variability is constrained by methodological limitations in detection, such as the failure to account for non-canonical secretion pathways and bioactive forms, as well as complex biomarker interactions—for instance, the modulatory countereffect of BDNF—which collectively undermine its reliability as a therapeutic target in comorbid conditions. There is an urgent need for prospective cohort studies in patients with depression and lung cancer to validate the dual efficacy of targeting the IL - 1β pathway (e.g., assessing whether anti-inflammatory interventions simultaneously improve psychological and oncological outcomes).

Furthermore, the development of a dynamic monitoring framework incorporating microenvironment-specific biomarkers is essential to bridge the gap between “well-established mechanisms and insufficient c IL - 1βIL-6, TNF-α, and IL - 1β serve as key inflammatory mediators in depression, each promoting tumor proliferation, metastasis, and metabolic reprogramming through activation of shared signaling pathways such as NF-κB, MAPK, and PI3K/AKT. However, their clinical relevance exhibits notable divergence: although elevated levels of IL - 6 and TNF-α show relative consistency across depression and cancer contexts—correlating with disease stage and treatment response (e.g., reduction following SSRI therapy)—the evidence regarding IL - 1β remains markedly contradictory. While some studies report positive correlations between IL - 1β levels and both depression severity and cancer progression, several meta-analyses have found no significant difference between patients with depression and healthy controls. These discrepancies may stem from methodological limitations in detecting non-canonical secretory pathways (e.g., exosome-mediated transport), context-dependent regulatory effects (such as counteracting interactions with BDNF), and heterogeneity across depressive subtypes.

Such inconsistencies underscore the limitations of relying on any single cytokine as a trans-diagnostic biomarker or therapeutic target. Future research should aim to integrate multidimensional markers—including inflammatory subtypes, neurotrophic factors, and metabolic profiles—and conduct well-designed prospective cohort studies to validate the dual therapeutic potential of targeting these pathways in patients with comorbid depression and cancer. A summary of the mechanism and clinical significance of IL-6, TNF-α, and IL-1β in cancer progression is provided in **Table 2**.

**Table 2:** Mechanism and clinical significance of IL - 6, TNF-α and IL - 1β in cancer progression.

Cytokines	Signaling pathway	Mechanism of cancer promotion	Prognosis and clinical association
IL-6	JAK-STAT3 MAPK/ERK PI3K- AKT	1)Promotion of Proliferation and Apoptosis Resistance 2)Promotion of Proliferation and Apoptosis Resistance 3)Establishment of a Positive Feedback Loop 4)Facilitation of Malignant Transformation 5)Dysregulation of Cell Cycle Control	1)In non-small cell lung cancer(NSCLC), elevated levels are associated with resistance to EGFR-TKI therapy and impaired NK/T-cell cytotoxicity (99). 2)In CRC, IL - 6 serves as a key regulator of CD8^+^ T cell infiltration. Among MSS/MSI-L subtypes, high IL - 6 expression is paradoxically associated with improved prognosis (100). 3) In high-grade serous ovarian cancer(HGSOC), high IL - 6R expression is linked to prolonged survival, suggesting a potential subset of patients who may benefit from targeted therapy (101). 4)In hepatocellular carcinoma(HCC), serum IL - 6 >10 pg/mL predicts increased mortality risk, and its combination with IL - 8 and CCL20 significantly enhances prognostic accuracy (102). 5)In bladder cancer, elevated serum IL - 6 is an independent risk factor for both overall and cancer-specific survival, and correlates positively with advanced tumor stage and adverse pathological features (103).
TNF-α	NF-κB PI3K- AKT ERK/JNK/p38- MAPK	1)Inhibition of Apoptosis 2)Sustained Activation Signaling 3)Promotion of Proliferation and Cell Cycle Progression 4)Induction of Angiogenesis 5)Enhancement of Invasion and Metastasis 6)Shaping an Immunosuppressive Microenvironment	1)In melanoma, TNF-α inhibitors may increase the risk of disease progression by suppressing immune surveillance, and when combined with PD - 1 inhibitors, are associated with a 15% increase in RECIST-defined progression rates (104). 2)In NSCLC, elevated levels of IL - 1, IL - 6, and TNF-α are observed, with TNF-α serving as an independent prognostic risk factor and showing a positive correlation with pain intensity (VAS score), supporting its utility in pain assessment (32). 3)In esophageal cancer, both TNF protein concentration and gene expression are significantly upregulated in tumor tissues, suggesting its potential role as a prognostic biomarker (105).
IL-1β	NF-κB JNK/p38- MAPK PI3K- AKT	1)Promotion of Angiogenesis 2)Enhanced Invasion and Metastasis 3)Inhibition of Ferroptosis 4)Remodeling of the Tumor Microenvironment	1)In pancreatic ductal adenocarcinoma (PDAC), accumulation of IL - 1β+ TAMs is associated with accelerated disease progression and reduced survival, while IL - 1β blockade delays tumor growth (111). 2)In breast cancer, IL - 1β drives metastasis by promoting myelopoiesis toward immunosuppressive neutrophils; its inhibition restores normal granulopoiesis and reduces metastatic burden (112). 3)In HCC, patients carrying the high-risk IL - 1β rs16944 AA genotype exhibit increased IL - 1β secretion, which independently correlates with elevated circulating tumor cells (CTCs), portal vein invasion, and shortened overall survival (113).

## 5 Effect of antidepressant drugs on cytokine levels

Antidepressants modulate the cytokine network through the neuroimmune axis, exhibiting marked drug-class specificity and dependence on baseline inflammatory status. Among SSRIs, sertraline significantly reduces serum levels of IL - 1β, IL - 6, and TNF-α in adolescents with depression (88). Fluoxetine, on the other hand, mitigates cardio-cerebral injury in individuals with myocardial infarction and comorbid depression by suppressing the TNF-α/TNFR/NF-κB pathway in macrophages, while also lowering pro-inflammatory cytokines such as IL - 17 and IFN-γ (89). Emerging evidence indicates that SSRIs may also enhance anti-tumor immunity by inhibiting the serotonin transporter (SERT) in T cells, thereby attenuating serotonin-mediated negative feedback and potentiating the cytotoxic response of CD8^+^ T cells—highlighting their broad regulatory potential across neural and immune systems (90).

Serotonin-norepinephrine reuptake inhibitors (SNRIs), such as duloxetine, significantly reduce levels of IL - 8, IL - 12, and IFN-γ, with baseline IL - 8 emerging as a predictor of treatment response (91). Notably, the norepinephrine-dopamine reuptake inhibitor (NDRI) bupropion exhibits a distinct immunomodulatory profile: after four weeks of treatment, patients show elevated levels of cytokines including IL - 1β, IL - 4, IL - 5, IL - 7, and IL - 8 compared to both pretreatment measures and healthy controls, alongside reductions in HDRS scores. A marked percentage increase was particularly observed in anti-inflammatory cytokines such as IL - 4, IL - 5, IL - 10, and IL - 13, suggesting that bupropion may actively promote an anti-inflammatory milieu rather than merely suppressing inflammation (92).

These findings illustrate that the immunomodulatory effects of antidepressants are not unidirectional but are instead characterized by multimodal and context-dependent complexity: while SSRIs and SNRIs generally suppress pro-inflammatory signals, bupropion appears to co-induce a broad cytokine response that includes anti-inflammatory mediators, ultimately fostering immune rebalancing.

However, significant limitations remain. Most evidence derives from peripheral blood measurements, which may not accurately reflect central immune activity; the causal relationship between cytokine changes and clinical improvement remains unclear; and the physiological and pathological implications of bupropion-induced cytokine elevations are not yet fully understood. Future research should integrate high-dimensional immune profiling and cell-specific mechanistic investigations to advance antidepressant therapy from a population-level paradigm toward personalized immunomodulatory strategies.

## 6 Clinical trials of IL - 6, TNF-α and IL - 1β pathway blockade in cancer and depression

A range of inhibitors targeting the IL - 6 signaling pathway—including monoclonal antibodies against IL - 6 or its receptor (e.g., tocilizumab, siltuximab), JAK inhibitors such as ruxolitinib, and STAT3-specific inhibitors like napabucasin—exhibit multifaceted potential in cancer therapy: they effectively suppress tumor growth, reverse the immune-inhibitory microenvironment, and synergize with immune checkpoint inhibitors to enhance anti-tumor immune responses (93). Notably, IL - 6-targeting antibodies such as sirukumab and siltuximab have demonstrated additional benefits in improving depressive symptoms among patients with rheumatoid arthritis (RA) and multicentric Castleman disease (MCD). However, these agents have not been systematically evaluated in individuals with MDD, nor in those experiencing major depressive episodes comorbid with conditions such as RA (94). Crucially, direct clinical evidence remains lacking regarding whether IL - 6 receptor blockade can yield dual improvements in both clinical symptoms and immune status among patients with cancer and comorbid depression. There is an urgent need for rigorously designed interventional studies targeting this specific population.

Infliximab, a monoclonal antibody targeting TNF-α, demonstrated no significant effect on primary tumor growth in a syngeneic BALB/c mouse model of 4T1 breast cancer; however, it reduced pulmonary metastasis incidence by 60%. This antitumor activity was linked to the suppression of TNF-α-mediated NF-κB and lysophosphatidic acid–autotaxin (LPA–ATX) signaling pathways, resulting in effective inhibition of distant metastasis (95).In the context of clinical depression management, a randomized controlled trial evaluated the efficacy of infliximab in 60 patients with treatment-resistant depression (TRD), using high-sensitivity C-reactive protein (hs-CRP >5 mg/L) as an inflammatory stratification marker. In the high-inflammation subgroup (hs-CRP >5 mg/L), infliximab treatment over 12 weeks led to significant reductions in HDRS scores, with notable improvements in core symptoms including anhedonia, anxiety, suicidal ideation, depressed mood, and psychomotor retardation. In contrast, among patients with low inflammation (hs-CRP ≤5 mg/L), placebo response surpassed that of the active treatment. Another randomized controlled trial involving 23 patients with ankylosing spondylitis (AS) and comorbid depressive symptoms showed that after 24 weeks of infliximab treatment, the Center for Epidemiologic Studies Depression (CES-D) scores decreased markedly from a baseline of 15.5 to 9.5, whereas scores in the placebo group remained around 18.0. This therapeutic benefit occurred independently of changes in CRP levels (96).At present, large-scale prospective trials are still needed to validate the efficacy of infliximab in populations with cancer and comorbid depression. Future research should integrate multidimensional immune profiling and mechanistic investigations to advance the development of precision immunomodulatory strategies.

In both chronic social defeat stress (CSDS) and LPS-induced rodent models of depression, serum levels of interleukin-1 receptor antagonist (IL - 1ra) were observed to be significantly elevated and showed a strong positive correlation with the severity of depression-like behaviors. Both models also induced a disrupted IL - 1ra/IL-1β ratio within the hippocampus. Further investigation revealed that chronic intracerebroventricular infusion of IL - 1ra not only completely prevented the emergence of depression-like behaviors induced by CSDS, but also reversed stress-associated reductions in hippocampal dendritic spine density and impairments in AMPA receptor (AMPAR)-mediated synaptic transmission. The underlying mechanism of this antidepressant-like effect was found to depend on activation of the cAMP response element-binding protein–brain-derived neurotrophic factor (CREB–BDNF) signaling pathway in the hippocampus, ultimately leading to enhanced synaptic function. These collective findings establish IL - 1ra as a highly promising novel therapeutic target for depression (97).It is noteworthy that antagonism of the IL - 1 signaling pathway also demonstrates therapeutic value in oncology. In a mouse model of colorectal cancer (CRC), administration of the IL - 1 receptor antagonist Anakinra effectively blocked the IL - 1β/IL-1R1 signaling axis. By inhibiting IL - 1β-driven activation of cancer-associated fibroblasts, it remodeled the immunosuppressive tumor microenvironment, thereby suppressing tumor growth and significantly prolonging host survival (98). These results suggest that IL - 1ra may have trans-diagnostic therapeutic potential, particularly offering a novel combinatory intervention strategy for cancer patients with comorbid depression. However, clinical translation will require addressing several challenges, including delivery efficiency, dissociation between central and peripheral effects, and disease-specific regulatory mechanisms.

In summary, biologics targeting inflammatory pathways such as those mediated by IL - 6, TNF-α, and IL - 1 exhibit considerable potential for intervening in depression and cancer comorbidity. Although preclinical and preliminary clinical evidence suggests that these agents may ameliorate both tumor progression and depression-like behaviors, there remains a conspicuous lack of high-quality interventional trials specifically designed for cancer patients with comorbid depression. Critical questions regarding the central versus peripheral mechanisms of action of these inhibitors, along with their safety profiles, blood-brain barrier penetrability, and long-term immunologic impacts, have yet to be adequately addressed. Future research must leverage precision immunophenotyping—using biomarkers such as CRP and IL - 6 levels—integrated with multi-omics and neuroimaging markers, to conduct mechanism-driven clinical trials. Only through such rigorous approaches can we truly bridge the gap from modulating inflammation to achieving integrated neuro-oncological therapy.

## 7 Conclusion

This review systematically delineates the mechanisms by which depression facilitates cancer progression through neural and systemic inflammation—centered on key cytokines including IL - 6, TNF-α, and IL - 1β—and activation of multiple oncogenic signaling pathways, thereby establishing a robust mechanistic framework for depression–cancer comorbidity.

However, clinical translation faces considerable challenges. First, the majority of evidence originates from animal or cellular models, and the profound heterogeneity and compensatory mechanisms within human tumor microenvironments limit the efficacy of single-pathway targeting strategies. Second, depression-associated inflammatory markers, such as IL - 1β, exhibit significant variability across populations and are influenced by regulatory factors like BDNF, undermining their reliability as universal biomarkers. Most importantly, although anti-inflammatory interventions—for instance, infliximab—have shown promise in inflammatory subtypes of depression (e.g., patients with elevated CRP), prospective interventional trials targeting populations with depression–cancer comorbidity are conspicuously absent. Thus, it remains unproven whether cytokine-targeted therapies can concurrently ameliorate both depressive symptoms and oncological outcomes.

Future research must extend beyond mechanistic description to identify operationally definable immune endotypes, develop integrated interventions targeting both central and peripheral inflammatory pathways, and, through rigorously designed clinical trials, evaluate the therapeutic value of combined anti-inflammatory, anti-tumor, and antidepressant strategies. Ultimately, such efforts will bridge molecular insights with precision clinical applications. ,
